# Supplementary material for: Causal Relationship between Aging and Anorexia Nervosa: A White-Matter-Microstructure-Mediated Mendelian Randomization Analysis
Source: Biomedicines. 2024 Aug 16;12(8):1874. doi: 10.3390/biomedicines12081874 (PMC11351342; doi:10.3390/biomedicines12081874)
Supplement: Supplementary file 1 [file biomedicines-12-01874-s001.zip › Supplementary Figure/Figure S8/Leaveoneout plot of MR analysis for TL to ubm-b-1587.pdf]

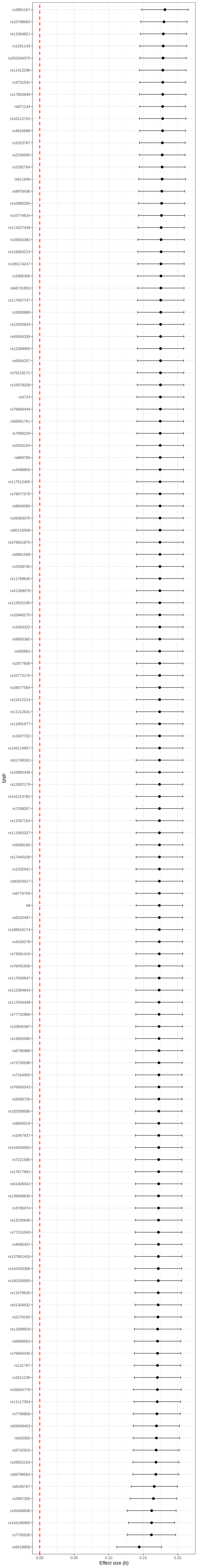
rs3891167
rs10768683
rs11584821
rs1291143
rs202034370
rs11412296
rs4731541
rs17803849
rs871134
rs10112752
rs4616688
rs1023767
rs2230590
rs2282764
rs611646
rs9878436
rs10905255
rs10774624
rs111527438
rs34550383
rs116863223
rs185174247
rs1985369
rs66731853
rs117407747
rs3093888
rs12925933
rs45604339
rs12369950
rs6054257
rs76219171
rs16978028
rs4724
rs76666449
rs56061761
rs7099229
rs2555104
rs869785
rs4498805
rs117512405
rs79977579
rs9940099
rs28363070
rs80116508
rs376641875
rs6881568
rs2538745
rs11769630
rs41269079
rs113525195
rs10840270
rs1003322
rs9955360
rs450962
rs2977608
rs10773176
rs28577594
rs12412214
rs11212631
rs11991877
rs1907702
rs145114957
rs61748181
rs10805346
rs12932179
rs141214782
rs7209057
rs11557154
rs111950327
rs9398196
rs17445108
rs1332941
rs80324517
rs6776756
All
rs8102497
rs188918174
rs4530278
rs73581419
rs78491606
rs117630647
rs112394943
rs117034449
rs77732866
rs10845387
rs13062095
rs6790988
rs73730598
rs7164950
rs76065543
rs2056726
rs182059586
rs9600019
rs1957937
rs144204502
rs7221585
rs17677991
rs61405042
rs139669835
rs3785074
rs13230646
rs77231040
rs4695407
rs137901416
rs142426306
rs150150565
rs11579626
rs41304832
rs2276182
rs11699829
rs6669563
rs75664430
rs131797
rs1611236
rs35640778
rs11117354
rs7790856
rs59409453
rs932002
rs5742915
rs28502153
rs56799554
rs8105767
rs2967355
rs35446936
rs143190905
rs7705526
rs9419958
SNP
0.00
0.05
0.10
0.15
0.20
Effect size (b)
